# Biometric analysis of furcation area of molar teeth and its relationship with instrumentation

**DOI:** 10.1186/s12903-024-04164-2

**Published:** 2024-04-10

**Authors:** Mun Mukherjee, Vineet Nair, Tanvi Phull, Ashish Jain, Vishakha Grover, Ahmed Babiker Mohamed Ali, Suraj Arora, Gotam Das, Saeed Awod Bin Hassan, Shan Sainudeen, Priyanka Saluja

**Affiliations:** 1 Independent Researcher, Kolkata, India; 2 Dr. R Ahmed Dental College and Hospital, Kolkata, India; 3 Department of Oral and Maxillofacial Surgery, Gian Sagar Dental College, Rajpura, Patiala India; 4 Department of Periodontology and Oral Implantology Dr.H.S.J. Institute of Dental Sciences, Panjab University, Chandigarh, India; 5 Department of Restorative Dental Sciences, College of Dentistry, King Khalid University, Abha, 61421 Saudi Arabia; 6 Department of Prosthodontics, College of Dentistry, King Khalid University, Abha, 61421 Saudi Arabia; 7 Department of Dentistry, University of Alberta, Edmonton, AB Canada

**Keywords:** Bone loss, Cervical enamel projection, Curette, Diagnosis, Enamel pearl, Furcation, Molar, Periodontitis, Prognosis, Root trunk length

## Abstract

The anatomy of furcation favours the bacterial retention and makes periodontal debridement as well as oral hygiene procedures difficult. Teeth that have lost attachment to a level of the furcation are said to have a furcal invasion or furcation involved.

Involvement of furcation in a multi-rooted tooth poses a very different type of clinical situation in terms of establishment of diagnosis, determination of prognosis and of course planning the treatment modality.The present study was carried out on 200 selected extracted human first and second permanent molar teeth based on a predefined criteria. Teeth with prosthetic crowns, fused or fractured roots, those not fully developed, grossly carious or heavily restored at the cementoenamel junction (CEJ) were excluded from the study. The morphology of the root trunk was recorded by measuring various dimensions of the root trunk,including furcal angle and root trunk volume was calculated by using a custom made special apparatus. The furcation areas were debrided with different types of curettes in the market in order to see how best the instrument could be maneuvered in the furcation area. The data so obtained was statistically analysed using SPSS version 22. The highest root trunk volume and the longest root trunk length were found to be in the maxillary second molar. 48.60% furcations didn’t allow instrument engagementof furcation area with standard area specific curettes. The proposal of inclusion of root trunk length (mm) is suggested in addition to classification of *FI* to have assess prognosis and appropriate treatment for of the involved tooth.

## Key messages

The complex anatomy of furcation area has important bearing on the root surface instrumentation and subsequently on the long term prognosis of periodontally treated teeth. There is a compelling need to understand the intricacies of this specific morphologic zone ,in context of periodontal instrumentation to enhance the effectiveness of local debridement, which is the cornerstone of standard periodontal therapy. Further, the addition of root trunk length & volume to existing classification systems of FI based on horizontal and vertical bone loss, shall prove a meaningful for providing better insights in to treatment planning and the prognostic evaluation of the periodontally involved teeth.

## Introduction

Periodontal disease is characterized by the loss of connective tissue attachment induced by the presence of periodontal pathogens within the gingival sulcus [1]. Once periodontal disease has been established, it progresses and further involves the furcation of multi-rooted teeth [2]. Compared to single-rooted tooth where there is no furcation, furcation in a multi-rooted tooth poses a very different type of clinical situation in diagnosis, prognosis and treatment plan [3].

Furcation may be defined as the anatomic area of a multi-rooted tooth where the roots diverge [4]. It has a complex anatomic morphology that may be difficult or impossible to debride during routine periodontal instrumentation and routine home care methods also may not keep the furcation area free of plaque [5, 6]. “Furcation involvement (FI) may be defined as the invasion of the bifurcation and trifurcation of multi-rooted teeth by periodontal disease” [7, 8].

The access to the furcation area is difficult both for the dentist and the patient and their treatment constitutes an enormous challenge. The treatment of teeth with FI ranges from thorough debridement to regenerative procedures and to extraction if the prognosis is hopeless [9, 10]. Phase I therapy i.e. local debridement of the involved tooth root is the first and most vital step of conventional periodontal therapy and has been documented as a pivotal element for the long-term success of periodontal treatment. The aim of the present study was to perform a retrospective biometric analysis of the furcation area of human molar teeth and understand the relevance of morphology of the furcation area in context of standard periodontal instruments used for local debridement of multirooted teeth.

## Materials and methods

**Fig. 1:**
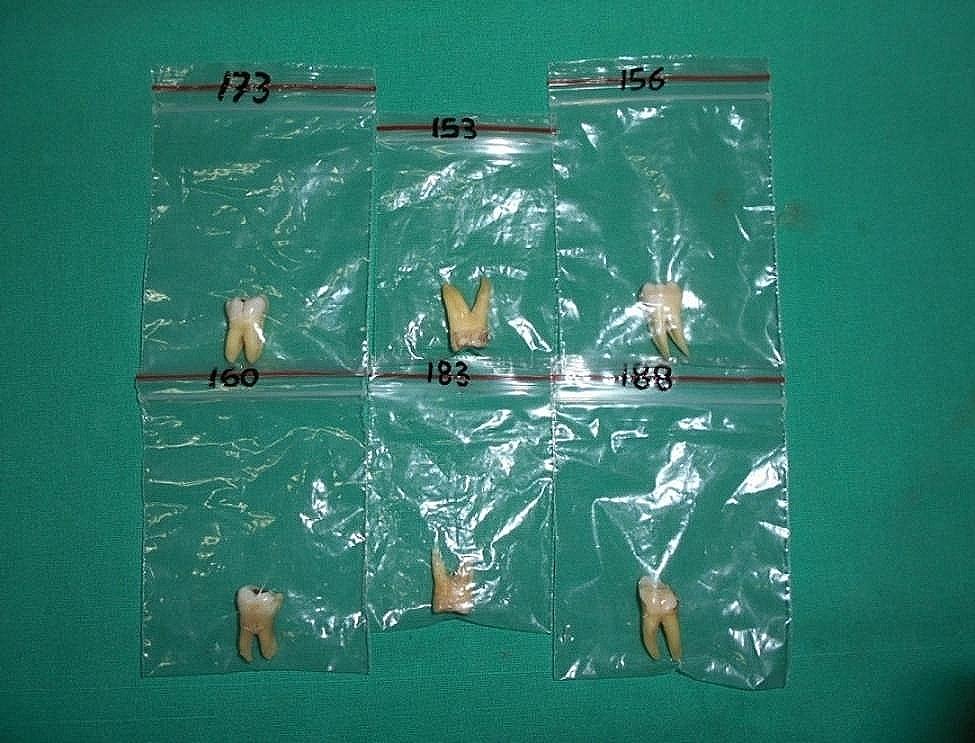
Teeth were numbered, labeled and preserved in pouches

The present study was carried out on 286 numbers of randomly selected extracted human first and second permanent molar teeth, collected from the outpatient department (OPD) of oral and maxillofacial surgery, in a dental institute. All the collected molars were washed in running tap water and were scrubbed with a hard bristled toothbrush and stored. After removal of the soft and hard deposits by ultrasonic scaling with a piezoelectric scaler (P5 Booster, Satelac) in-vitro, the teeth were kept in 5.5% sodium hypochlorite solution overnight. The teeth were again washed with water and dried. The tooth type was then determined, numbered, labeled, and preserved in pouches (Fig. 1).

Teeth with prosthetic crowns, fused or fractured roots, and those not fully developed, grossly carious or heavily restored at the cementoenamel junction (CEJ) were excluded from the study. Eighty-six molars were thus rejected and the remaining 200 molars were found to be in equal distribution as follows- maxillary first molar (*n* = 50), maxillary second molar (*n* = 50), mandibular first molar (*n* = 50) and mandibular second molar (*n* = 50). Thereafter, the teeth were again scrutinized thoroughly with a magnifying glass for final inspection on or below the CEJ.

**Fig. 2:**
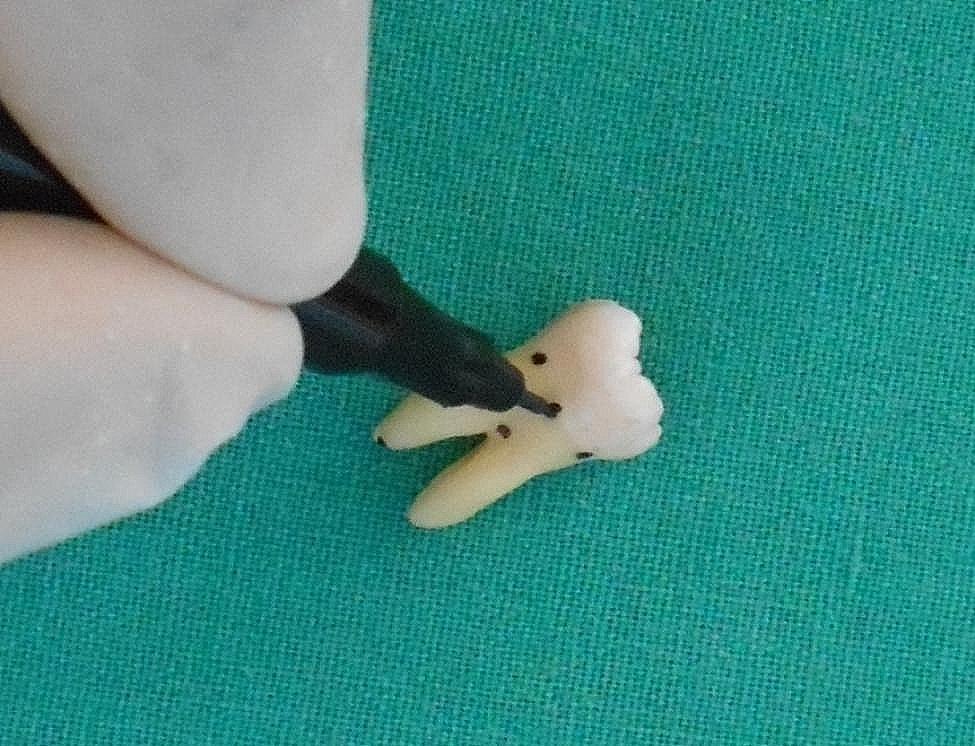
Marking of CEJ, point of division of roots at the furcation and the root apex

Examination of the site- with the help of a magnifying loupe (Surgitel, 2.5x), three locations- the anatomical location of CEJ, the point of division of the roots at the furcation and the root apex were identified and marked with a 0.5 mm black marker pen (Fig. 2). Simultaneously they were examined for anatomical variations like cementoenamel projections, enamel pearls and suitably noted.

Determination of root trunk volume- Setting up of the apparatus- A four cm long glass cylinder was placed in the middle of a 10 cm wide petri dish (now called apparatus herein after). The apparatus was placed in the working area alongside the electronic weighing balance CAS Model ME 310 (Maximum weight = 310 gm; difference = 0.001 gm).

**Fig. 3:**
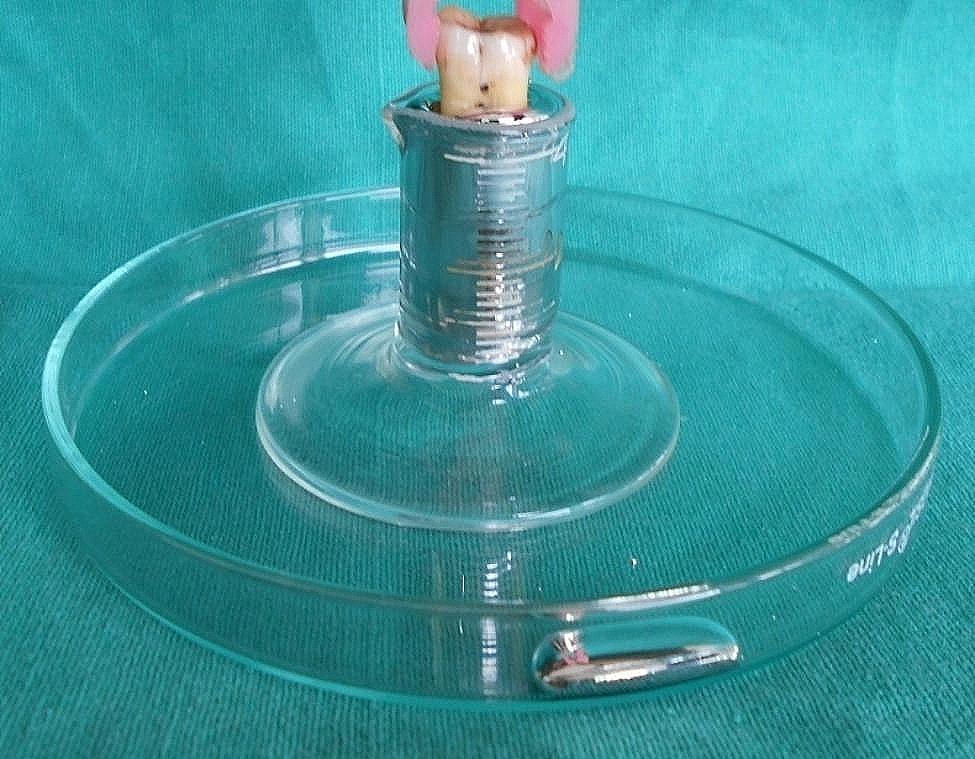
Tooth immersed until the furcation

**Fig. 4:**
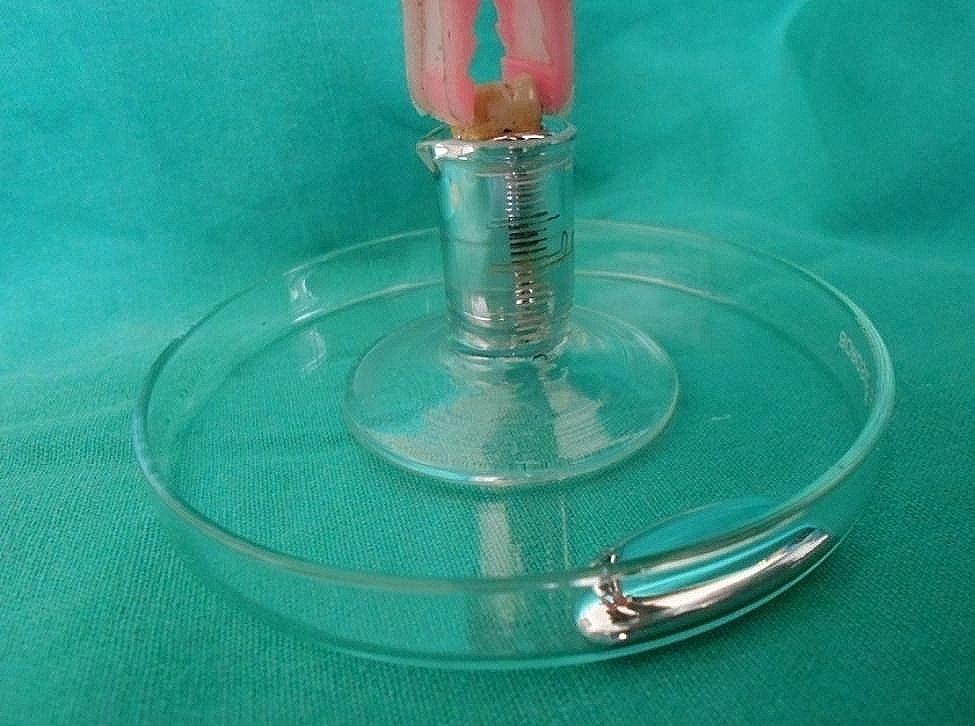
Tooth immersed until the CEJ

Mercury from the storage container was poured slowly and cautiously into the measuring cylinder until a drop of mercury came out on the petri dish of the apparatus. The mercury on the petri dish was then cleared and then the apparatus along with the mercury was weighed and recorded (first reading). The apparatus was then carefully removed from the weighing balance. Each tooth sample was taken and introduced into the mercury until its furcation (Fig. 3) and was checked with a magnifying glass. Therefore, some droplets were expelled out of the measuring cylinder onto the petri dish. The expelled mercury was cleared and the measuring cylinder with the remaining mercury was put back on the weighing balance and the weight was recorded again (second reading). The apparatus with the remaining mercury was brought back to the working station and the same tooth was again introduced into the mercury until the CEJ (Fig. 4), was simultaneously checked with a magnifying glass. Some mercury was again expelled out onto the petri dish. The mercury was cleared and the apparatus along with the remaining mercury was weighed and recorded (third reading). All the measurements were done by a single examiner to avoid intra-examiner error.

### Calculation of volume

Volume of the root cones (until furcation) = (first reading - second reading)**/**density of mercury.

The volume of root trunk = (second reading - third reading) /density of mercury.

(The density of mercury was taken as 13.534/cm^3^)

### Measurement of furcation angle

**Fig. 5:**
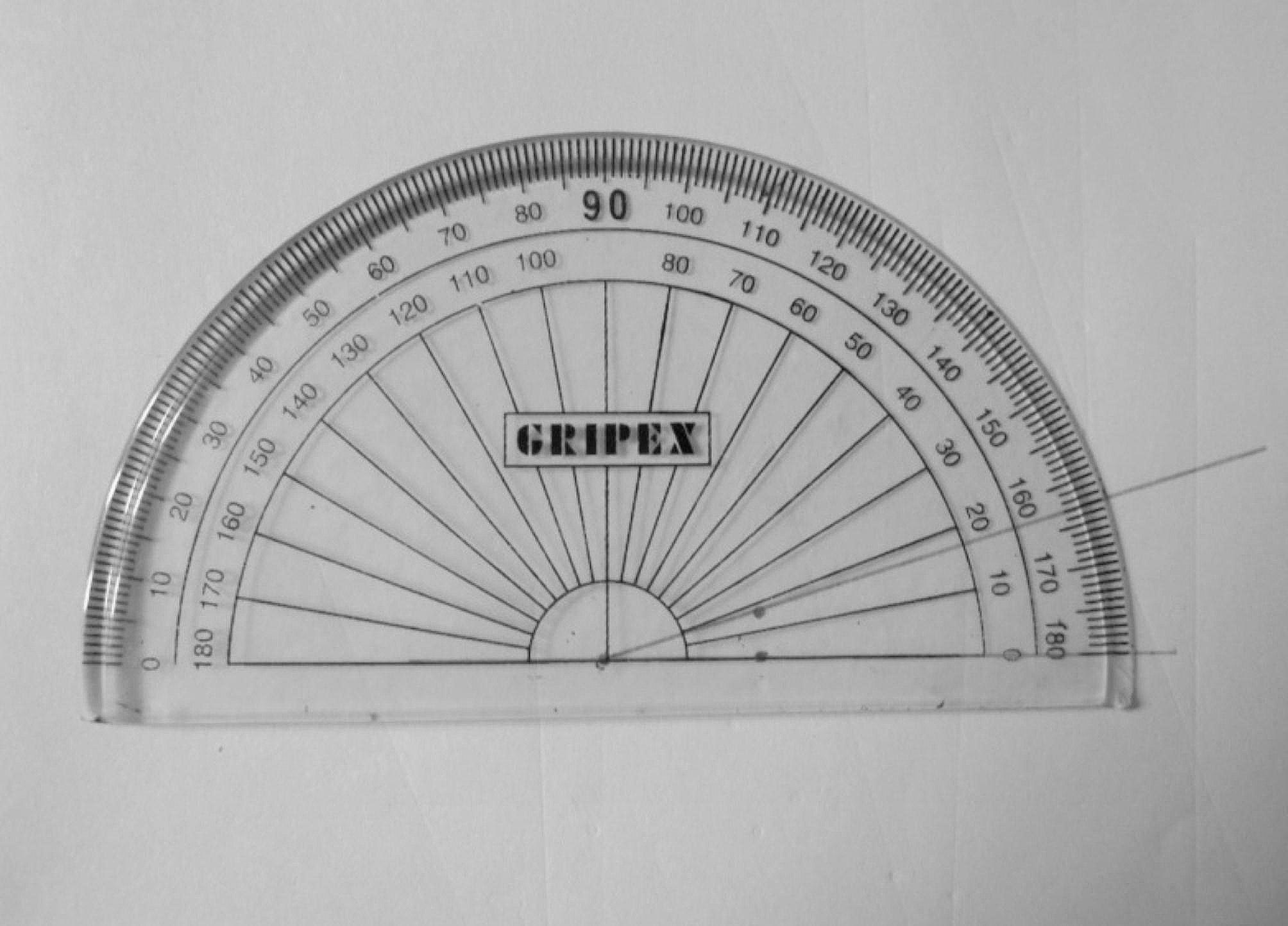
Determination of the angle of furcation

The furcation angle of each tooth was measured with a protractor which was placed on the working table. A horizontal line was drawn on the working table. The tooth was placed on the line to mark the CEJ on the line. To measure the angle between two roots, one root was superimposed on the line and the apex was marked on the line. Another apex was marked on the working table. So, the angle formed between the CEJ, and the two apexes was measured (Fig. 5).

**Fig. 6:**
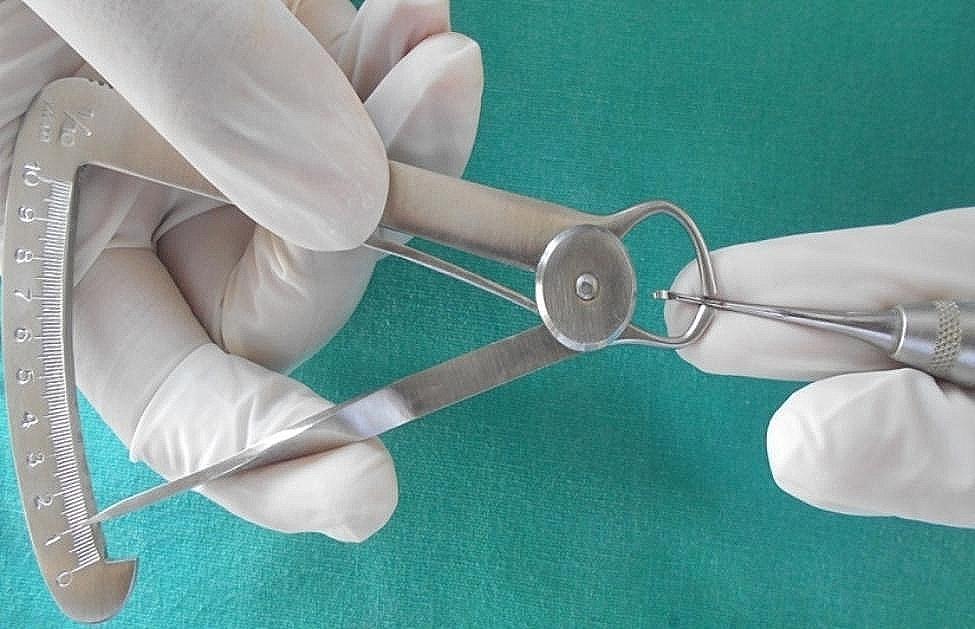
Blade – face width

In order to record the linear dimensions of the furcation, a standardized scale was developed by measuring the dimensions of the diameter of different sizes of steel needles and then testing the furcation entrance by introducing standardized gauge. The blade-face dimension (Fig. 6) of the curette was measured and noted. Introduction of curette was then done in the teeth. The recordings were suitably tabulated. Each of the mandibular molars (having two furcation entrances) and the maxillary molars (having three entrances) were tested for the linear dimension by introducing the standardized gauge carefully and recording properly.

### Measurement of tooth root dimension

**Fig. 7:**
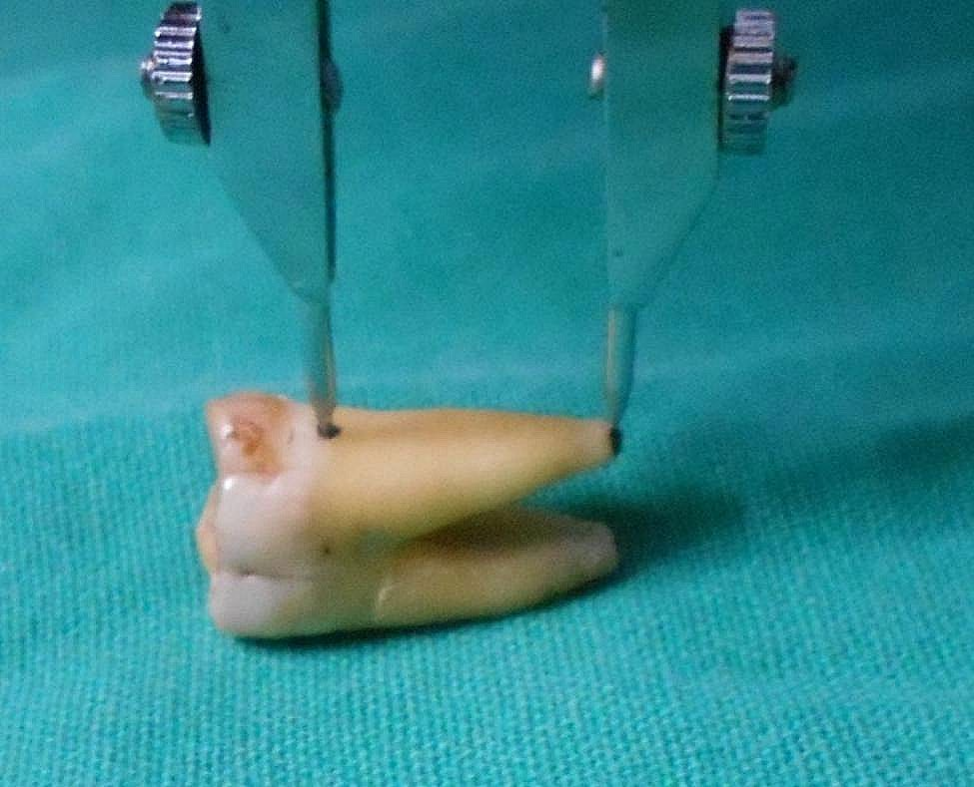
Determination of root length

**Fig. 8:**
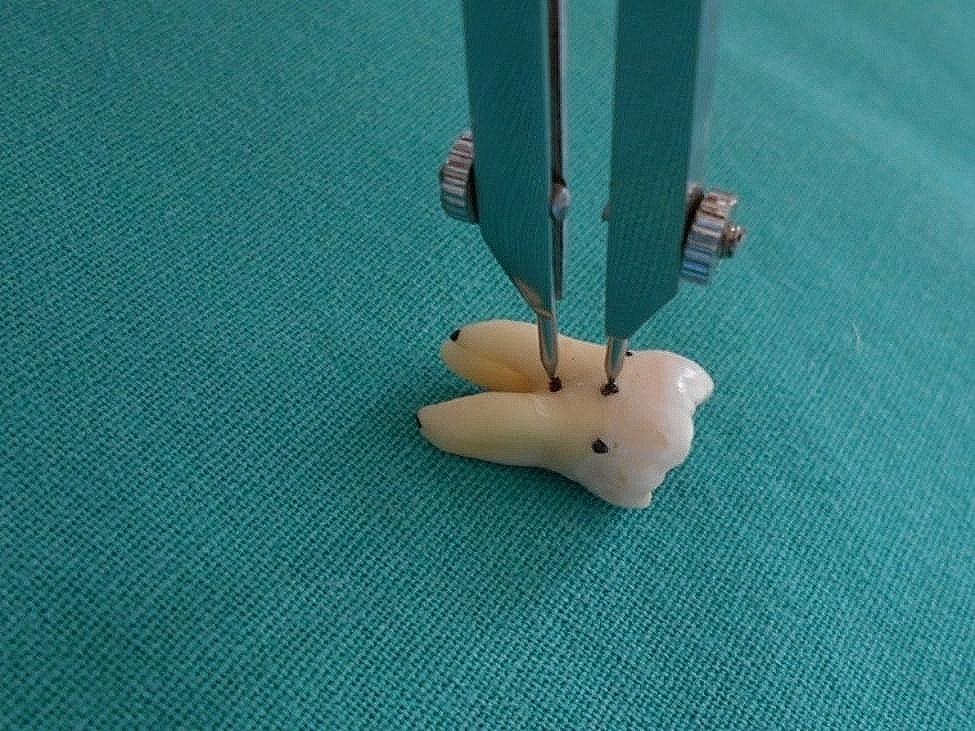
Determination of root trunk length

- Root length- With the help of a divider the length of root apex to CEJ (Fig. 7) was traced, which was then placed on a metallic scale for measurement and recorded. This was done for all the three roots of the upper molars and both roots of the lower molars. Each reading was taken carefully and checked again with a magnifying glass.

b. Root trunk length- Similarly the root trunk length was traced with the help of a divider from CEJ (Fig. 8) to the furcation point which was then placed on a metallic scale for measurement and recorded. The same was checked with a magnifying glass.

### Statistical analysis

Statistical analysis was performed with the help of the latest SPSS software. Descriptive statistical analysis was performed to prepare different frequency tables and to calculate the means with corresponding standard errors. Chi–square test was applied as the measure of association. One way analysis of variance (ANOVA) followed by Tukey’s Test was performed with the help of critical difference (CD) or least significant difference (LSD) at 5% (CD_5_) and 1% level (CD_1_). t-test was used to compare the means. *p* < 0.05 was taken to be statistically significant.

## Results

The measurements obtained from the 200 molars were arranged into tables to portray the volume of root cones and trunk (Table 1), furcation angle (Tables 2 and 3), root trunk length in maxillary molars (Tables 4, 5, 6 and 7), and in mandibular molars (Tables 8, 9 and 10).

**Table 1:** Volume of root cones (cu.cm.) and volume of root trunk (cu.cm) SD- standard deviation

Molar	Volume of root cones (mean ± sd)	Volume of root trunk (mean ± sd)
Maxillary first (*n* = 50)	0.51 ± 0.12	0.22 ± 0.08
Maxillary second (*n* = 50)	0.44 ± 0.17	0.23 ± 0.11
Mandibular first (*n* = 50)	0.35 ± 0.13	0.21 ± 0.09
Mandibular second (*n* = 50)	0.33 ± 0.15	0.20 ± 0.07

**Table 2:** Distribution of buccal, mesiopalatal and distopalatal angles in maxillary molar

Molars	Buccal angle (in degree) (mean ± sd)	Mesiopalatal angle (in degree) (mean ± sd)	Distopalatal angle (in degree) (mean ± sd)
Maxillary first (*n* = 50)	27.46 ± 8.23	40.66 ± 6.36	43.78 ± 8.43
Maxillary second (*n* = 50)	21.05 ± 6.19	36.74 ± 7.97	36.45 ± 7.82

**Table 3:** Distribution of lingual angle and buccal angle in mandibular molar

Molar	Lingual angle (in degree) (mean ± sd)	Buccal angle (in degree) (mean ± sd)
Mandibular first (*n* = 50)	27.43 ± 7.49	28.45 ± 8.76
Mandibular second (*n* = 50)	25.55 ± 6.57	26.70 ± 8.37

**Table 4:** Distribution of root trunk length of maxillary molars

Molar	Buccal root trunk length (mm) (mean ± sd)	Distal root trunk length (mm) (mean ± sd)	Mesial root trunk length (mm) (mean ± sd)
Maxillary first (*n* = 50)	3.65 ± 0.63	4.07 ± 0.98	4.09 ± 0.71
Maxillary second (*n* = 50)	3.87 ± 0.91	4.22 ± 0.99	4.85 ± 1.12

**Table 5:** ANOVA table for buccal root trunk length (maxillary molars) (*p* < 0.01)

Source	D.F	Sum of squares	Mean sum of squares	F	p
Between groups	1	1.21	1.21	1.975	> 0.05
Within group	98	60.03	0.613	-	-
Total	99	61.24	-	-	-

Critical difference (CD) values: CD_5_ = 0.47 and CD_1_ = 0.59

**Table 6:** ANOVA table for distal root trunk length (maxillary molars) (*p* < 0.01)

Source	D.F	Sum of squares	Mean sum of squares	F	p
Between groups	1	0.590	0.590	0.603	> 0.05
Within group	97	95.035	0.979	-	-
Total	98	95.626	-	-	-

Critical difference (CD) values: CD_5_ = 0.52 and CD_1_ = 0.72

**Table 7:** ANOVA table for mesial root trunk length (maxillary molars) (*p* < 0.01)

Source	D.F	Sum of squares	Mean sum of squares	F	p
Between groups	1	14.18	14.18	16.29	< 0.01
Within group	97	84.45	0.87	-	-
Total	98	98.63	-	-	-

Critical difference (CD) values: CD_5_ = 0.56 and CD_1_ = 0.72

**Table 8:** Distribution of root trunk length of mandibular molars

Molar	Lingual root trunk length (mm) (mean ± sd)	Buccal root trunk length (mm) (mean ± sd)
Mandibular first molar	3.77 ± 1.10	3.72 ± 1.11
Mandibular second molar	3.79 ± 0.71	3.55 ± 0.88

**Table 9:** ANOVA table for lingual root trunk length (mandibular molars) (*p* < 0.01)

Source	D.F	Sum of squares	Mean sum of squares	F	p
Between groups	1	0.01	0.01	0.0116	> 0.05
Within groups	98	84.65	0.864	-	-
Total	99	84.66	-	-	-

Critical difference (CD) values: CD_5_ = 0.42 and CD_1_ = 0.56

**Table 10:** ANOVA table for buccal root trunk length (mandibular molars) (*p* < 0.01)

Source	D.F	Sum of squares	Mean sum of squares	F	p
Between groups	1	0.7225	0.7225	0.7246	> 0.05
Within groups	98	97.705	0.996	-	-
Total	99	98.4275	-	-	-

Critical difference (CD) values: CD_5_ = 0.42 and CD_1_ = 0.51

## Discussion

Furcation is an important area in terms of assessment of bone loss, diagnosis, prognosis and treatment planning for teeth with FI. Therefore, the current has been undertaken to study the furcation area morphology and its clinical relevance for periodontal instrumentation.

The methods of determining the root trunk length in previous studies were based on using the electric caliper micrometer by Hou and Tsai (1997) [11] and Dababneh et al. (2011) [12]; contracer machine by Rios et al. (2002) [13] and radiographs by Hou et al. (2005) [14]. Our study made an attempt to determine the volume of the root trunk in addition to its vertical dimension to have an idea of the volumetric concept of the root trunk part and for this a new method of the assessment of volume of the root trunk using mercury was adopted. This liquid (mercury) seems to yield the right volume of trunk. The other liquids of low surface tension like alcohol, water, oil could not be employed because of the low density and impart out the wettability.

The present study found average root lengths of 12.79 and 13.10 mm for maxillary first and second molars respectively and 13.45 mm and 13.35 mm for the first and second mandibular molars respectively. This data is in accordance with the study of Tarnow et al. (1984) [15]. According to Wheeler (2003) [16] the average length of the roots of maxillary first molars was 12.5 mm and 11.5 mm for the second molars, while for mandibular molars it was 14 mm and 13 mm for the first and second molar respectively. In our study, t-test showed that the mean volume of root cones for maxillary first molar was significantly higher than that of maxillary second molar (*p* ≤ 0.01) but no significant difference was found for root trunk (*p* > 0.05). No significant difference was found between mandibular first molar and mandibular second molar for mean volume of root cones or mean volume of root trunk (*p* > 0.05, Table 1). The consideration of determining volume was to know the amount of periodontal supporting structure within which the tooth is embedded and its subsequent destruction due to the disease process. The length of the root trunk alone is not sufficient to conceive the idea of a peri-cemental area surrounding the root whose destruction takes place in case of disease progression from CEJ apically.

The maxillary bone is mainly cancellous type and its posterior area is mainly made up of thin trabecular bone with wider marrow space than the anterior area. It can be well conceived that compared to the anterior part of the dental arch where biting is done, the posterior part is concerned with crushing of the food which requires a greater force and probably this being the reason to withstand the greater amount of force, the root surface area is made greater, by dividing the roots usually into two or three. It is seen that the angle of divergence in upper molars with special reference to the palatal root becomes detrimental to the health of the tooth by precipitating recession, but the mandibular teeth do not have such a problem of being subjected to recession. The anteroposterior display of roots does not cause such a problem.

Clinically the angle of divergence has got its importance by the fact that it offers more biomechanical anchorage to its underlying bone. Moreover, higher divergence of roots offers better instrumentation in the furcation area. That higher divergence offers better facilitation of instrumentation in the furcation area was reported by Johnson et al. (2013) [17]. In our study, the t-test showed that the mean buccal angle, mesiopalatal angle, and distopalatal angle of maxillary first molars were significantly higher than that of the maxillary second molars (*p* < 0.01, table-2). The present study found that the highest angle of divergence was the distopalatal angle (43.78 degrees between distobuccal and palatal root) of maxillary first molars and the lowest angle recorded was the buccal angle (21.05 degrees between mesiobuccal root and the distobuccal root) of the maxillary second molar (Table 2). Both the mean lingual and the buccal angles of the mandibular first molars were higher than that of the mandibular second molars and it was statistically significant (*p* ≤ 0.05) (table- 3). The narrow difference in the angles in lower molars may be due to the difference in the methodologies adopted. This study presents a simpler way of measuring the angle in contrast to the computerized mathematical derivation by Johnson et al. (2013) [17].

Statistical analysis (ANOVA) of our study showed that there was neither any significant difference (*p* > 0.05) between buccal root trunk length of the maxillary first molar and maxillary second molar (Table 5) nor on the distal side (table- 6) though there was a significant difference (*p* < 0.01) between the same on the mesial side (table-7). Again, there was neither any significant difference between lingual root trunk length of mandibular first molar and mandibular second molar (*p* > 0.05, table- 9) nor on the buccal side (*p* > 0.05, table- 10). The root trunk length of the second molar has been found to be greater than the first molar and statistically significant. Different molars vary in trunk diameters; therefore, the length of destruction cannot only predict the amount of loss of surrounding supporting structures.

On physical examination of extracted 200 molars with the aid of magnifying loupe and/or magnifying glass, the present study did not reveal any enamel pearl. However, Martos et al. (2009) found 15 enamel pearls out of 177 molars [18]Cervical enamel projections (CEP) noted and classified as per Masters and Hoskins (1964) [19] were grade III in one tooth whereas the large majority of 160 samples showed grade I i.e. the teeth with a discrete enamel projection towards the furcation. Martos et al. (2009) found highest frequency (n 80 = 28.6%) of grade I while 17 teeth (6%) and 33 teeth (12%) fell under grade II and III respectively [18]. Bhusari et al. (2013) reported a total of 112 (11.9%) CEP out of 944 molars examined [20]. Out of these 112 teeth, 82 (8.68%) were reported to be grade III which in contrast was frequent and 15 (1.5%) each categorized under grades II and I. The inclusion of third molars by Bhusari et al. in their study can be attributed to the difference in the results.The CEP as a predisposing factor for the initiation of periodontal disease is well established [18, 21].However, it can hardly be diagnosed early by routine clinical and radiographic examinations. It is tedious from the clinical perspective to ascertain the presence of CEP in the absence of a periodontal pocket [22]. This fact reinforces the need for adequate oral hygiene in the treatment protocol in the presence of CEP. The most common (85.37%) grade I CEP has got the least clinical importance. In contrast, CEP of grade II or III has an immense clinical relevance in the progression of the disease and its subsequent entry into the furcation [23–25].

The available literature reports the difficulty of doing the periodontal procedures with the help of proper instruments. The standard curette used in this study was area specific (Hu-Friedy) and the blade face width accounted for 1 mm. This blade could not be introduced into the furcations having a linear dimension less than 1 mm or having an angle of less than 30 degrees (in this study it is 243 out of 500 i.e., 48.60% furcations didn’t allow instrument engagement). 227 (45.40%) furcations belonging to the angle 30–60 degrees having linear dimension 0.75-1 mm could allow the curette blade but meager engagement of the instrument was not sufficient for a better and efficient clinical manueverity. Merely, 6 furcations in this study (1.2%) could offer a convenient access to the furcation area with the introduction of specific curette and facilitated its proper instrumentation and thus it was evident that furcation area offers a real difficulty in its treatment and management in day to day clinical practice.

We have discussed in previous sections that the furcation area presents a complex anatomy. It affects not only periodontal treatment,but almost every dental treatment procedureimplied in this zone ae.g. access cavity preparation during root canal treatment, subgingival margin placement during crown and prosthesis preparation and also in various periodontal treatment modalities (i.e. root planing).If the root trunk length is more, the height of the pulp chamber (i.e., the distance from the roof to the floor of the pulp) will increase and there will be more difficulties in access cavity preparation and more sophisticated instruments need to be used. More coronal dentin will be available if the root trunk length is longer and that will provide the better ferrule effect in case of subgingival margin placement. More pulp volume will be available if the root trunk length is longer. So, it could be hypothesized that more hypersensitivity would be experienced by the patients during extensive root planing procedure.

The availability of studies on furcation and its anatomy have been minimal and the classifications thus established lacked in some perspective or the other. Ever since, Glickman (1958) [26]. started his work, there were rampant changes, modifications, and amendments of his classification. In the beginning only the horizontal nature of bone destruction was addressed to [3, 8, 27–37], though later the vertical loss component was also considered [38–44].Unfortunately, these classification systems established till date still lack a vital dimension i.e., the root trunk length. The vertical bone loss is considered from the point of furcation in apical direction only and not from CEJ to apical direction. The tooth root is submerged in the alveolar bone up to the CEJ and whatever the alveolar bone surrounding the root gets destroyed should be accounted for, in determining the exact classification. In the classification systems,where the tissue destruction is assessed from the point of furcation, the part of the alveolar bone surrounding the root trunk remains undetermined objectively. For a more precise assessment of the severity of FI,the root trunk area and volume should also be accounted for ,in addition to horizontal and verticalcomponent of tissue destruction. A new improvised and much elaborate classification system has been proposed of periodontal and peri-implant diseases and conditions in 2018 [43], yet the determinants to classify FI has been largely unchanged,so far.

Hou and Tsai (1995) [44] attempted for the first time to classify the root trunks into three different types A, B, C and emphasized to incorporate this into the present-day system of furcation classification. This division of root trunks has been found to be gross and did not consider millimeter-wise vertical destruction. For a tooth to be stable in its bony socket, a millimeter wise assessment of vertical alveolar bone loss should be considered as an objective parameter. Our study underscores the importance of the dimensions of root trunk length in the morphologic assessment of furcation area of human teeth and emphazies an objective evaluation of this area, in addition to the present day systems to evaluate the FIs for appropriate therapeutic and prognostic assessments for periodontally diseased individuals. for enhancing succesful treatment outcomes.

## Conclusion

With in the limitations of the study, the findings highlight the complexity of the furcation area assessments and suggests the practical limitations for the accessibility of FIs with standard periodontal instruments for the purpose of local debridement of periodontally involved teeth. Further, inclusion of objective assessment of root trunk length or volume to prevailing systems of classification of FI might improve the prognostic and therapeutic results in these cases. The current study paves the path for future investigations on larger number of samples to delve further into the anatomic considerations, relevant to therapeutic modalities for more predictable treatment outcomes in FIs.

## Data Availability

The data presented in this study are available upon request from the corresponding author.
